# A future of automated image contouring with machine learning in radiation therapy

**DOI:** 10.1002/jmrs.365

**Published:** 2019-12-19

**Authors:** Price Jackson, Tomas Kron, Nicholas Hardcastle

**Affiliations:** ^1^ Department of Oncology Sir Peter MacCallum University of Melbourne Melbourne Victoria Australia; ^2^ Department of Physical Sciences Peter MacCallum Cancer Centre Melbourne Victoria Australia; ^3^ Centre for Medical Radiation Physics University of Wollongong Wollongong NSW Australia

## Abstract

Automated image contouring is showing improvements in efficiency for a number of clinical tasks in radiotherapy. While atlas segmentation has proven moderately beneficial, the next generation of algorithms based on convolutional neural networks is already pointing to improvements in precision and efficiency. This work provides a broad overview of the benefits of machine learning when applied to these tasks.

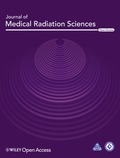

The article, ‘Implementing User‐defined Atlas‐based Auto‐segmentation for a Large Multi‐Centre Organisation: the Australian Experience’, provides a helpful insight into the utility of automated contouring algorithms in real‐world clinical use.^1^ The authors provide careful consideration to identifying potential applications of automated segmentation based on frequency of use and contouring difficulty. While some of their findings indicate that atlas‐based algorithms are yet to replace the human observer in contouring tasks such as soft tissue in the pelvis, it does set the stage for assessing and deploying progressively improved tools in the future. This includes how to quantify the utility of automatically generated planning contours and use traditional matching scores (Dice Coefficient) relative to time required for manual correction.

While the authors point to mixed results in terms of overall accuracy with atlas methods – a finding that depends on anatomical region – one noteworthy analysis relates to interpreting efficiency gains and user feedback. Although over half of thorax and head and neck structures required either no or minor change, this translated into an overall efficiency gain in the order of only 20%. A detailed analysis shows that the majority of structures required some modification before being fit‐for‐use in treatment planning and in addition to the time required to make corrections, one must consider the time and disruption to review each structure individually. In terms of computational efficiency, even modern atlas contouring techniques require significant overhead to identify best‐match cases and perform non‐rigid registration to match individual labels. So while these processes may be automated, they are not instantaneous and attempts to improve accuracy by incorporating hundreds or thousands of training cases may become impractical due to losses in computational efficiency. It is becoming apparent, however, that the shortcomings of this generation's automated contouring algorithms will be addressed as technology transitions to the use of convolutional neural networks (CNNs).

These neural networks, often interchangeably termed Deep Learning with the capabilities of modern computing, utilise a series of pattern recognition stages. The early layers of the network detect basic image features such as edges and specific intensity ranges through filter convolution. Subsequently, intermediate layers convolve the output of the prior stages, which over multiple stages enables the network to interpret objects in terms of shape, size and configuration with respect to nearby structures. In practice, this allows CNNs to learn the appearance of objects in a manner that is similar to the human eye where recognition involves detecting a number of relevant characteristics.

As atlas‐based methods perform best with subject data that matches incoming cases in terms of overall appearance, it is advisable to generate centre‐specific atlases based on the local scanning equipment. Curating these datasets with representative patients that include all potential tissues‐of‐interest represents another potentially costly overhead. While deep learning techniques certainly benefit from site‐specific image data which corresponds with the appropriate imaging conditions (reconstruction kernels, imaging field‐of‐view, slice spacing, etc.), the generalisability may be partially addressed through considered data augmentation. The appearance of a single dataset may be modified through random degrees of local blurring, edge‐enhancement and added noise to mimic broad variety of scan conditions. While this increases the amount of time required to train the algorithm, the final model remains efficient to deploy and should be resilient to a variety of scan parameters. This reduces the reliance on centre‐specific models and should better accommodate changes to protocols or equipment due to hardware upgrade.

While CNNs can require significant computational overhead for model training – the optimisation of filters and layer weights over sometimes several days – the deployment, or inference, of a prepared neural network on each patient to be segmented is highly efficient and enables flexibility to develop separate models for a number of specific tasks. In the work by Hu et al.^1^, the authors note that optimal performance was observed instances where the selected atlas case closely matched the patient's anatomy. This highlights one of the challenges with atlas‐based contouring methods. If a patient's anatomy does not conform to one of the typical cases included in the training set, it is expected that the global contouring accuracy across many structures will suffer. Additionally, because all regions are derived from one or only a handful of closely matched cases, many structures may be well adapted, but this will likely be to the detriment of others. In the presented atlas‐segmentation work, the authors have considered this shortcoming and were able to increase the performance of the female thorax algorithm by directing it to select two reference cases which best matched either the organs or the lymph nodes and muscle; effectively doubling the processing time required. However, one advantage of CNN approaches is that even if one network is used for all structures in a region, the contours from each case will all be learned independently of each other. That is, ultimately any contour is based on the combined knowledge of all included training cases. Further, because of the rapid processing of trained models, it is still relatively efficient to employ separate CNNs for each volume‐of‐interest which ensures that the selection of features to delineate subtle structures are not overwhelmed by larger or higher contrast regions.

In just the last 3‐4 years, there has been a rapid shift towards development in these computational methods for medical imaging and with particular focus in radiation therapy.^2^, ^3^ Because the training process employs statistically based optimisation, it is tolerant to a certain fraction of noisy or poorly curated training data if, on balance, this permits the inclusion of a larger training cohort.^4^ Radiation therapy planning is an ideal candidate to use AI‐based contouring as routine practice generates high‐quality structures, though ones that may vary in terms of precision or style. So while Hu et al. employed atlas methods using the careful selection and review of approximately 30 cases per modality, with machine learning‐based methods it is viable to incorporate hundreds of cases using existing structure data – perhaps after a cursory review – and the output of the model will represent the best weighted prediction from the much larger cohort.

Looking beyond the automation of routine clinical work, the ability to generate accurate organ contours on‐the‐fly makes it feasible to collect measurements that would typically be considered too time consuming to acquire outside of dedicated research studies. In radiation therapy, this should enable better analysis of dose‐response relationships to non‐critical structures; those that may not normally be contoured or difficult to perform with high precision. There is scope to use machine learning methods to contour primary tumour volumes, a task cannot be addressed through atlas segmentation.^5^ Further, automated contouring that is robust to image variations may facilitate delineation and subsequent analysis of anatomical, dosimetric and organ function changes on images acquired during treatment course, such as repeat cone‐beam CTs or MRIs. Machine learning techniques have the potential to be extremely adaptable when given well‐conditioned challenges.

Many factors point towards a future of medical imaging that takes advantage of advanced computation to improve or speed up existing processes. It should enable better standardisation as the ‘style’ of an AI model will be the same when deployed across different hospitals. As clinicians become familiar with automation in medicine, these methods should help to collect more precise and frequent measurements from medical imaging. If the latter comes to fruition, this will necessitate increased focus on ensuring well‐curated clinical outcome data are available to provide clinical correlates to any imaging biomarkers. Rather than simply removing humans from the picture, automation should enable more careful oversight by simplifying mundane tasks, help to reveal a deeper understanding of the underlying biology, and free up clinicians’ time to focus on the patient.

## Funding Information

It may be worthwhile to state that Tomas Kron and Nicholas Hardcastle both "receive funding from Varian Medical Systems for an unrelated project."
